# Efficaciousness of Low Affinity Compared to High Affinity TSPO Ligands in the Inhibition of Hypoxic Mitochondrial Cellular Damage Induced by Cobalt Chloride in Human Lung H1299 Cells

**DOI:** 10.3390/biomedicines8050106

**Published:** 2020-05-02

**Authors:** Nidal Zeineh, Nunzio Denora, Valentino Laquintana, Massimo Franco, Abraham Weizman, Moshe Gavish

**Affiliations:** 1 The Ruth and Bruce Rappaport Faculty of Medicine, Technion Institute of Technology, Haifa 31096, Israel; nidalz1988@gmail.com; 2 Department of Pharmacy–Pharmaceutical Sciences, University of Bari “Aldo Moro”, 70125 Bari, Italy; nunzio.denora@uniba.it (N.D.); valentino.laquintana@uniba.it (V.L.); massimo.franco@uniba.it (M.F.); 3 Institute for Chemical and Physical Processes (IPCF)-CNR SS Bari, Via Orabona 4, 70126 Bari, Italy; 4 Research Unit at Geha Mental Health Center and Laboratory of Biological Psychiatry at Felsenstein Medical Research Center, Petah Tikva 4910002, Israel; weizmana@gmail.com; 5 Sackler Faculty of Medicine, Tel Aviv University, Tel Aviv 6997801, Israel

**Keywords:** translocator protein (TSPO), CoCl_2_, mitochondrial membrane potential, reactive oxygen species (ROS), cell viability, cell death, lung cancer cell line

## Abstract

The 18 kDa translocator protein (TSPO) plays an important role in apoptotic cell death, including apoptosis induced by the hypoxia mimicking agent cobalt chloride (CoCl_2_). In this study, the protective effects of a high (CB86; Ki = 1.6 nM) and a low (CB204; Ki = 117.7 nM) affinity TSPO ligands were investigated in H1299 lung cancer cell line exposed to CoCl_2_. The lung cell line H1299 was chosen in the present study since they express TSPO and able to undergo programmed cell death. The examined cell death markers included: ATP synthase reversal, reactive oxygen species (ROS) generation, mitochondrial membrane potential (Δψm) depolarization, cellular toxicity, and cellular viability. Pretreatment of the cells with the low affinity ligand CB204 at a concentration of 100 µM suppressed significantly (*p* < 0.05 for all) CoCl_2_-induced cellular cytotoxicity (100%), ATP synthase reversal (67%), ROS generation (82%), Δψm depolarization (100%), reduction in cellular density (97%), and also increased cell viability (85%). Furthermore, the low affinity TSPO ligand CB204, was harmless when given by itself at 100 µM. In contrast, the high affinity ligand (CB86) was significantly effective only in the prevention of CoCl_2_–induced ROS generation (39%, *p* < 0.001), and showed significant cytotoxic effects when given alone at 100 µM, as reflected in alterations in ADP/ATP ratio, oxidative stress, mitochondrial membrane potential depolarization and cell death. It appears that similar to previous studies on brain-derived cells, the relatively low affinity for the TSPO target enhances the potency of TSPO ligands in the protection from hypoxic cell death. Moreover, the high affinity TSPO ligand CB86, but not the low affinity ligand CB204, was lethal to the lung cells at high concentration (100 µM). The low affinity TSPO ligand CB204 may be a candidate for the treatment of pulmonary diseases related to hypoxia, such as pulmonary ischemia and chronic obstructive pulmonary disease COPD.

## 1. Introduction

In this study the hypoxic toxic agent cobalt chloride (CoCl_2_) was used to induce hypoxic death in H1299 lung cancer cells. Hypoxia is a state in which the oxygen supply to a tissue within an organ is compromised [1]. Decreased oxygen levels or tension in a tissue results in hypoxic damage due to altered cellular signaling networks. Hypoxia may occur both in physiological conditions such as climbing to high altitudes, as well as in pathological conditions such as myocardial ischemia, chronic obstructive pulmonary disease (COPD), and obstructive sleep apnea [2]. Hypoxia affects cellular homeostasis, which may lead to reactive oxygen species (ROS) production, elevated vasoconstrictive substances, increased expression of pro-inflammatory cytokines, and increased intercellular and vascular cell adhesion molecules [3,4,5,6,7,8,9]. Impaired protein homeostasis in the muscle system is a well-known condition in COPD patients [10,11,12].

CoCl_2_ mimics hypoxic state when applied to cell cultures [13,14,15,16,17,18,19]. Previous studies in neural-derived PC12 cells, demonstrated that CoCl_2_ might induce apoptotic cell death via the P38-MAPK-caspase 3 pathway [20]. CoCl_2_ interferes with heme production from protoporphyrin IX (PPIX) [19,21]. PPIX acts as an endogenous ligand for the mitochondrial 18 kDa translocator protein (TSPO) [22]. It was shown that decreased ROS levels due to TSPO knockdown lead to accumulation of PPIX and consequently increased heme production [23]. The hypoxia mimicking effect of CoCl_2_ is related to its stabilizing effect on the α-subunit of HIF (hypoxia inducible transcription factor), in addition to inhibition of cytochrome-C oxidase subunit 4 (COX4) precursor processing and enhancement of its degradation [16].

**Scheme 1:**
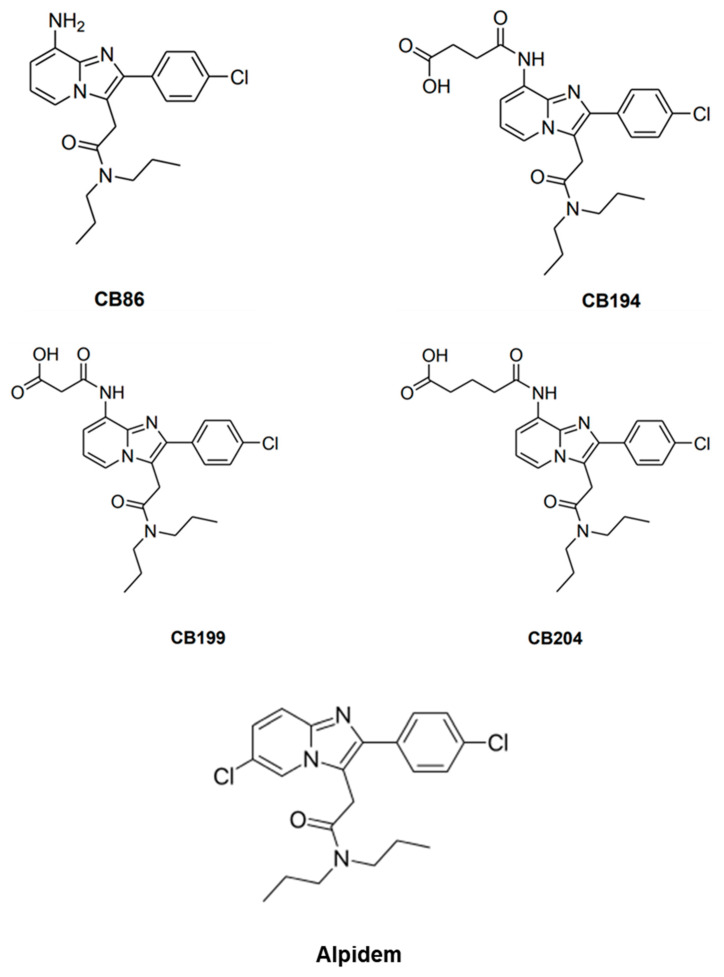
Molecular structure of CB86, CB194, CB199, CB204, and Alpidem assayed in the present study.

Mitochondria are involved in some of the hypoxia-induced cellular injury mechanisms, and it acts as an energy source, as well as a source for metabolic signals, cellular proliferation, inflammation, and intrinsic cell death [24]. The TSPO located in the outer mitochondrial membrane plays an important role in the mitochondrial apoptotic processes [25,26,27,28,29], along with other cellular roles [30,31,32]. A previous study has indicated the role of TSPO in CoCl_2_-induced apoptotic cell death. TSPO knockdown in U118MG glioblastoma cells, demonstrated the important role of TSPO in apoptosis induced by CoCl_2_, including processes such as: Δψm depolarization, cardiolipin peroxidation, and ROS generation. Administration of the TSPO ligand PK 11195 inhibited partially a sequence of TSPO-related processes relevant to CoCl_2_-induced cell death reminiscent of the effects of TSPO knockdown determined in the same study [22]. In the present study, TSPO ligands characterized by a 2-phenyl-imidazo [1–a] pyridine nucleus (Scheme 1) that express various affinities were investigated (Table 1). The goal of the current study was to examine, for the first time, the protective effect of two novel TSPO ligands (CB86 and CB204), in the attenuation of the hypoxic effect of CoCl_2_ in H1299 lung cancer cell line. The two ligands differ in their affinity to TSPO: CB204 has low affinity (Ki = 117.7 nM) and CB86 has high affinity (Ki = 1.6 nM) [33]. Among the few examples known of TSPO ligands containing hydrophilic groups, there is the 8-amino imidazopyridine CB86 ligand. In a previous study it was evaluated whether further polar substituents or ionizable functional groups could be introduced on the amino group of CB86 [33]. Data indicated that introduction of a -COOH group led to a significant decrease in affinity to TSPO, as observed for the compound CB204. Furthermore, in addition to the structure-activity relationship studies, reported by some of us, highlighting the main physicochemical factors eliciting the binding of imidazopyridines to TSPO, the pharmacological profile of these compounds by measuring their modulatory effects on the GABA_A_ receptors was also explored [33].

CB86 and CB204 were chosen in the present study for their diverging affinities to the TSPO. This choice was prompted by previous findings with other TSPO ligands, presenting low to moderate affinity, that showed efficacy regarding cellular protective effects and without cellular toxic activity. In contrast, high affinity TSPO ligands can induce cellular toxic effects and conspicuous lethal effects at relatively high concentrations [34,35,36]. These previous studies were conducted on microglia, astrocytic, neuronal, and cancer cells and in animal models [35,37,38]. A previous review of numerous cell types reported that classical high affinity TSPO ligands show lethal effects at high concentrations (typically > 50 µM), but protective effects at low concentrations [39]. A subsequent experimental research reported that indeed in a paradigm of astrocytic cells challenged with ammonia, the classical high affinity TSPO ligands (PK 11195, Ro5 4864 and FGIN-1-27) induced cell death at concentrations above 50 µM, but were protective at the nM range [40]. Thus, the hypothesis of the present study was that the high affinity TSPO ligand (CB86 in Scheme 1) would show cytotoxic effects at a concentration of 100 µM, while the low affinity TSPO ligand with a comparable structure (CB204 in Scheme 1) would show cellular protective effects at the same concentration of 100 µM. We applied this to a paradigm of cells vastly different from the cells regularly used by us (lung cancer cells vs. brain cells). We attempted to confirm or disprove previous findings on the relationship between the affinity of ligands to TSPO and their cytotoxic or protective effects. Furthermore, the question was whether these cellular effects are specific for brain cells, or valid also for other types of cells as well, in our case lung cells. The present report provides new data since: (1) The TSPO ligands in the current study were not used in the previous studies; (2) low affinity and high affinity TSPO ligands based on a common structural framework are compared in one paradigm, so they can reliably represent their unique pharmacological properties; and (3) another type of cells (lung cells) are used, while in the previous similar studies brain derived cells were used. The present study was designed to provide indication whether the previous findings on the effects of TSPO ligands on brain derived cells can also be discerned with novel TSPO ligands when applied to other types of cells, and thus are not restricted to the cells of brain origin (microglia, astrocytes, and neuronal cells). Thus, the present attempted to verify and unify the image suggested by the scattered information of previous studies. We chose H1299 lung cells because they represent peripheral respiratory mitochondrial-relevant system, express TSPO and can undergo programmed cell death when exposed to cytotoxic agents [41].

Thus, the correlation between TSPO ligand affinity and their protective effects in a putative TSPO-associated hypoxic cellular model was evaluated in the present study.

## 2. Materials and Methods

### 2.1. Study Design

In this study, the H1299 lung cancer cells from human origin were used. The maintenance of the cells was performed according to the American Type Culture Collection (ATCC) instructions, as follows: Culture medium consisted of RPMI (high glucose, without L-glutamine and sodium pyruvate), supplemented with 10% Fetal Bovine Serum (FBS), 2% glutamine, and gentamycin (50 mg/mL). The serum-deprived medium contained similar components, just with 2.5 mL FCS (0.5%), instead of 50 mL (10%). According to a previous study, hypoxic damage due to exposure to toxic agent (e.g., cigarette smoke) demonstrated an association between TSPO and apoptotic cell death. Since the conditions needed were the existence of TSPO and the possibility to induce apoptosis via TSPO, H1299 fulfilled these criteria, and were chosen in the current study [41].

The cells were incubated at 37 °C in 5% CO_2_ until 80–90% confluency was reached. Initially, pretreatment of the cells with the TSPO ligands: CB86, CB194, CB199, CB204, and Alpidem, at increasing concentrations was performed 24 h prior to incubation with CoCl_2_ for another 24 h. According to the collected results, the high affinity CB86 and the low affinity CB204 TSPO ligands were chosen for the proceeding experiments.

### 2.2. CoCl_2_ Exposure and Its Treatment with TSPO Ligands

According to previous studies, the CoCl_2_ at a concentration of 0.5 mM was used in our experiments [22,42,43].

Treatment consisted of 24 h of TSPO ligand pretreatment prior to the subsequent 24 h simultaneous exposure to CoCl_2_ and TSPO ligands/or vehicle. In more detail, pretreatment of the cells was conducted using DMSO (1%) (vehicle) in serum-deprived medium for the control groups, and ligand-containing serum-deprived medium for the experimental groups. After 24 h of pretreatment, the medium of the control groups and the ligand-containing groups was replaced by the same medium, but in the “experimental groups” it contained also 0.5 mM CoCl_2_. Then, the groups of cells were incubated for another 24 h (the treatment), as previously described [43,44,45]. The pretreatment groups included: vehicle and vehicle plus ligand groups. For treatment, the groups included: Vehicle, vehicle plus ligand, vehicle plus CoCl_2_, and vehicle plus ligand plus CoCl_2_ groups. Following this subsequent exposure to CoCl_2_ for 24 h, the cells were collected and processed. According to a previous study using U118MG cells [22], dose response analysis with increasing CoCl_2_ concentrations was performed. It was shown that at concentrations of 0.3–0.5 mM cell death levels increased by 10–25%, while at concentrations above 0.6 mM cell death elevated by 40% to 90% [22]. In accord with the previous study [22], the 0.5 mM concentration of CoCl_2_ was selected for the current experiments, in order obtain reversible and treatable cellular toxic effects. The dose response curve for H1299 cells exposed to CoCl_2_ was very similar to the one described by us previously on U118MG cells [22].

### 2.3. ADP/ATP Ratio

White 96-well plates were used to measure the levels of ADP and ATP. After pretreatment of cells in medium with and without CB86 and CB204, followed by CoCl_2_ exposure, the ADP/ATP ratio was measured and calculated, according to the manufacturer’s protocol (MAK135; Sigma-Aldrich, St. Louis, MO, USA), as previously described [46]. Luminescence levels of the ratio between ADP and ATP were measured by ELISA using Infinite M200 Pro plate reader (Tecan, Männedorf, Switzerland), according to the instructions of the manufacturer. 

### 2.4. Reactive Oxygen Species (ROS) Levels

ROS/superoxide detection assay kit (Abcam, Cambridge, UK) was used to measure ROS and superoxide levels according to the manufacturer’s instructions. Following treatment of the cells as described above, they were washed using 1X wash buffer followed by application of 100 μL/well of ROS/superoxide detection solution and then incubated in dark for 60 min. ELISA with Infinite M200 Pro plate reader (Tecan, Männedorf, Switzerland) was used with standard fluorescein (excitation 488 nm and emission 520 nm) and rhodamine (excitation 550 nm and emission 610 nm) filter sets, and fluorescence levels were measured, according to the instructions of the manufacturer.

### 2.5. Depolarization of the Mitochondrial Membrane Potential (ΔΨm)

Mitochondrial membrane potential (ΔΨm) depolarization was assayed using the dye JC-1(5,5′,6,6′-tetrachloro-1,1′,3,3′-tetraethylbenzimidazolylcarbocyanine-chloride), as described previously [22]. JC-1 is a marker for mitochondrial function and cell health. Intact cells characterized by high Δψ_M_, the cationic lipophilic JC-1 dye selectively enters the mitochondria, forms aggregates and thus reversibly changes color from green to red inside the mitochondria (orange-red fluorescence emitting at 590 nm wavelength). In case of Δψ_M_ depolarization, monomers of JC-1 remain in the cytoplasm, and these monomers emit at 527 nm (green fluorescence). Thus, Δψ_M_ depolarization is expressed by decreased red/green ratio. 

The cells were trypsinized and collected by centrifugation (660× *g*, 5 min, 4 °C). The proton ionophore carbonyl cyanide was used as a positive control, as previously described [22]. The samples were incubated in 400 μL of diluted JC-1 dye (1/500) for 30 min. Then, the cells were centrifuged (660× *g*, 5 min, 4 °C), the pellets then resuspended in 500 μL of PBS and transferred into FACS tubes. The mean fluorescence intensity (MFI) was measured using FACS device, and the results were analyzed using FlowJo (10th version, FlowJo LLC, Ashland, OR, USA). In the calculation of the mitochondrial depolarization the basal 590/527 ratio was subtracted. This is an assay demonstrating cell number-wise, namely, the percentage of the measured cell population presents mitochondrial depolarization sufficient to prevent JC-1 entry and conversion to red emitting aggregates.

### 2.6. Cellular Cytotoxicity Assay (LDH)

Cellular cytotoxicity levels were measured by the Cytotoxicity Detection Kit (Roche Pharmaceuticals, Basel, Switzerland), which measures the LDH enzyme levels in the medium. LDH enzyme is released from the cells when the cell membrane is disrupted in case of necrosis and late apoptosis [47].

Cytotoxicity levels were measured by an absorbance at 492 nm wavelength and a reference wavelength of 620 nm, according to the manufacturer’s protocol. ELISA with spectrophotometer Zenyth 200 (Anthos, Eugendorf, Austria) was used for measurements and the results were calculated and normalized (calibrated) according to the formula given by the manufacturer.

### 2.7. XTT Based Colorimetric Assay

Cell viability was measured using XTT cell viability assay kit (Abcam, Cambridge, UK), according to the manufacturer’s protocol. In this assay, in viable cells the 2,3-bis [2-methoxy-4-nitro-5-sulphophenyl]-2H-tetrazolium-5-carboxylanilide inner salt (XTT) is reduced by mitochondrial dehydrogenases, and this reaction leads to an orange formazan product. This assay measures cell metabolism as an indication of cell viability. The spectrophotometer Zenyth 200 (Anthos, Eugendorf, Austria) was used to assess optical density (OD) as reflected by absorbance at 492 nm wavelength and a reference wavelength of 620 nm (ELISA technology).

### 2.8. Cell Density

Photographs from wells of a 96-well plate with the various experimental groups were taken using a Basler scA1400–30gm camera, a CCD camera with Sony ICX285 CCD sensor (Essen Bioscience, Ann Arbor, MI, USA). The confluency of the cells was measured using the high throughput, continuous live cell imager Incucyte Zoom HD/2CLR System (Essen Bioscience, Ann Arbor, MI, USA) to assess the effects of CoCl_2_ exposure on the density of the cells. The same number of cells were used in each assay for all wells. To ensure this, plating was done from one tube with cell suspension by collecting equal amounts from the tube for each well. In numbers, 40,000 cells per cm^2^ were seeded and then the cells were cultured till they reached 80–90% confluency.

### 2.9. Statistical Analysis and Data Presentation

Relative changes (%) were used for comparisons among assays. GraphPad prism (GraphPad Software, San Diego, CA, USA) was used for statistical analysis. The results were expressed as mean ± SEM. One-way analysis of variance (ANOVA) was performed followed by Bonferroni’s post hoc test for multiple comparisons as appropriate. *p* < 0.05 was considered as statistically significant.

## 3. Results

### 3.1. Screening of TSPO Ligand Efficacy in the Protection of Cellular Viability

**Figure 1:**
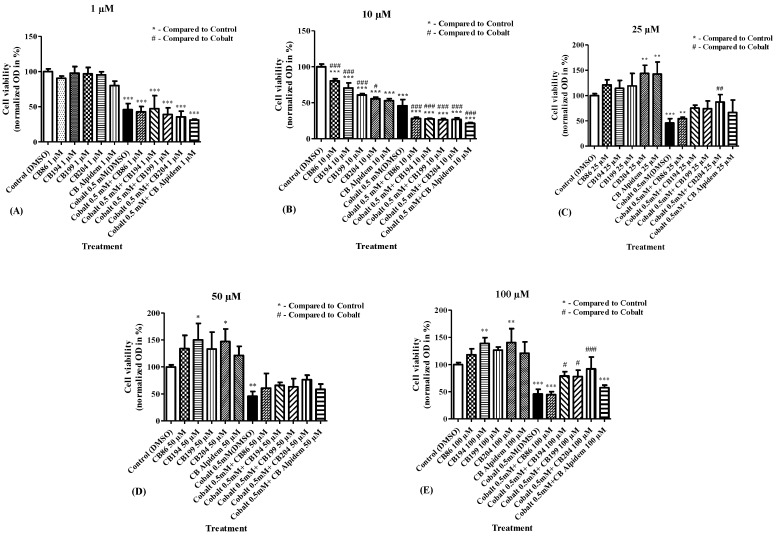
Determination of the ligand efficacy in inhibition of CoCl_2_-induced decrease in cellular viability (XTT assay). Cell viability was assessed following pretreatment with TSPO ligands at various concentrations of 1 µM, 10 µM, 25 µM, 50 µM, and 100 µM (**A**–**E**). Results are expressed as mean ± SEM (*n* = 5 replicates in each group). ^#^ and * *p* < 0.05, ^##^ and ** *p* < 0.01, ^###^ and *** *p* < 0.001.

Assessment of the efficacy of the TSPO ligands in the prevention of CoCl_2_ cytotoxicity was determined using a cellular viability assay (XTT) with different TSPO ligands CB86, CB194, CB199, CB204, and alpidem, at concentrations of 1 µM, 10 µM, 25 µM, 50 µM, and 100 µM for each of them (Figure 1A–E). It was demonstrated that the high affinity CB86 ligand and the high affinity alpidem, did not protect against the cytotoxicity caused by CoCl_2_ (0.5 mM), while the low affinity CB204 achieved significant protective capacity by 76% at a concentration of 25 µM (Figure 1C) and by 85% at 100 µM (Figure 1E). In addition, the low affinity CB194 and CB199 ligands displayed significant protection by 61% and by 59%, respectively, against CoCl_2_ cytotoxicity only at a concentration of 100 µM (Figure 1E). According to these data (Figure 1), the high affinity ligand CB86 and the low affinity ligand CB204 at a concentration of 100 µM were chosen for further investigation of the potential effects of the affinity of the TSPO ligands in the protection from CoCl_2_-induced cellular damage.

In general, going from high affinity to low affinity TSPO ligands (Table 1) was associated with larger protective effect (Figure 1).

### 3.2. Dose–Response Analyses for the Efficacy of CB86 and CB204 Ligands in the Prevention of CoCl_2_-Induced Decrease in Cellular Viability

**Figure 2:**
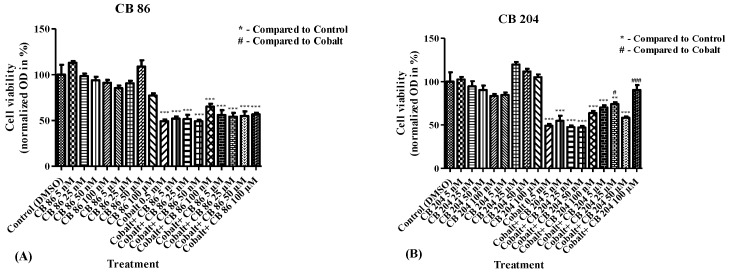
Dose response analysis of CB86 and CB204 ligands in inhibition of cellular viability decrease induced by CoCl_2_. XTT assay was performed to measure the levels of cellular viability following exposure to CoCl_2_ after pretreatment with (**A**) CB86 and (**B**) CB204 at increasing concentrations. Results are expressed as mean ± SEM (*n* = 5 replicates in each group). ^#^ *p* < 0.05, ^##^ and ** *p* < 0.01, ^###^ and *** *p* < 0.001.

To verify the indications described in Figure 1, the modulatory effect of CB86 and CB204 on cell viability following exposure to CoCl_2_ was assessed. The range of concentrations of these two ligands was expanded, from 1 µM to 100 µM (Figure 1), and from 5 nM to 100 µM (Figure 2), to ensure that no major concentration-dependent effects were overlooked. Dose–response assessment was performed for CB86 and CB204 using cellular viability assay (XTT). Pretreatment with increasing concentrations of CB86 did not inhibit the cytotoxic effect of 0.5 mM CoCl_2_ at any tested concentration (Figure 2A). In contrast, the low affinity TSPO ligand CB204 at concentrations of 25 µM and 100 µM prevented 50% and 81% of the cellular damage, respectively (Figure 2B). At 100 µM, CB204 caused maintenance at control levels, i.e., cell viability was not significantly different from vehicle-exposed cells. Figure 1 and Figure 2 show that 100 µM is the optimal concentration for CB204 capacity to provide protection from CoCl_2_-induced cell death.

### 3.3. ADP/ATP Ratio

**Figure 3:**
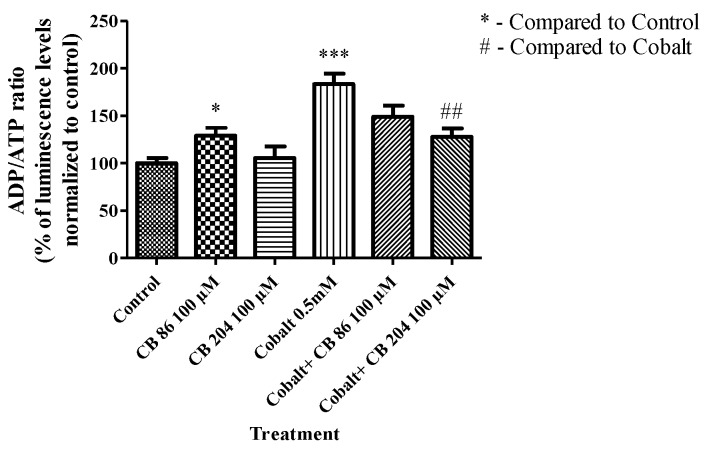
The ability of TSPO ligands to attenuate CoCl_2_-induced ATP synthase reversal. ELISA was performed to measure the alterations in ADP/ATP ratio with and without pretreatment with the TSPO ligands. Results are expressed as mean ± SEM (*n* = 5 replicates in each group). * *p* < 0.05, ^##^ *p* < 0.01, and *** *p* < 0.001.

Following CoCl_2_ exposure for 24 h, the ADP/ATP ratio significantly increased by 84% (*p* < 0.001) as compared to the control group (absolute level of ADP/ATP luminescence ratio was 0.1). This increase was not attenuated significantly by pretreatment with CB86 at a concentration of 100 µM (49%; *p* > 0.05), while pretreatment with CB204 at a concentration of 100 µM significantly attenuated (67%; *p* < 0.01) the increase in ADP/ATP ratio, as compared to the CoCl_2_-exposed group (Figure 3).

**Figure 4:**
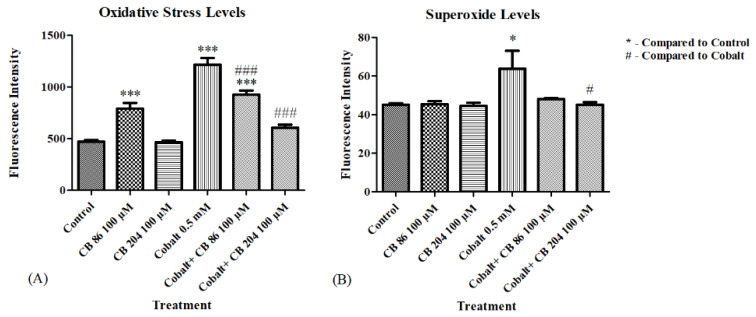
The protective effect of the TSPO ligands in prevention of reactive oxygen species (ROS) elevation. ELISA was performed to evaluate the levels of oxidative stress and superoxide levels. (**A**) Represents the oxidative stress levels, and (**B**) Represents the superoxide levels, with and without pretreatment with TSPO ligands. Results are expressed as mean ± SEM (*n* = 6 replicates in each group). ^#^ and * *p* < 0.05, ^###^ and *** *p* < 0.001.

Regarding ADP/ATP ratio, CB86 appears to have a dual effect: slightly cytotoxic by itself, but protective from the cytotoxic effects of CoCl_2_. Reminiscent of the effects on ROS levels described in Section 3.4 (Figure 4).

### 3.4. Oxidative Stress and Superoxide Levels

ROS levels were measured following CoCl_2_ exposure of CB86 and CB204 pretreated cells. Significant elevation in ROS levels was detected following pretreatment of the cells with CB86 ligand alone, while no elevation occurred following pretreatment with the TSPO ligand CB204 alone. CoCl_2_ induced a significant elevation in oxidative stress levels by (158%; *p* < 0.001) as compared to the control group. Pretreatment with CB86 at a concentration of 100 µM significantly prevented (39%; *p* < 0.001) of the CoCl_2_-induced increase in ROS generation, however remained significantly higher than the control (*p* < 0.001). In contrast, pretreatment with CB204 at a concentration of 100 µM significantly prevented 82% of the CoCl_2_-induced elevation (at the control range; *p* < 0.001; Figure 4A). In a second assay, superoxide levels were not elevated in response to the incubation with both ligands alone. CoCl_2_ exposure for 24 h induced a significant elevation (41%; *p* < 0.05) as compared to the control group, while pretreatment with CB86 did not prevent significantly the elevation in superoxide levels (*p* > 0.05; Figure 4B). CB204 was more effective in preventing this elevation (actually to control range; *p* < 0.05; Figure 4B).

### 3.5. Depolarization of the Mitochondrial Membrane Potential (ΔΨm)

**Figure 5:**
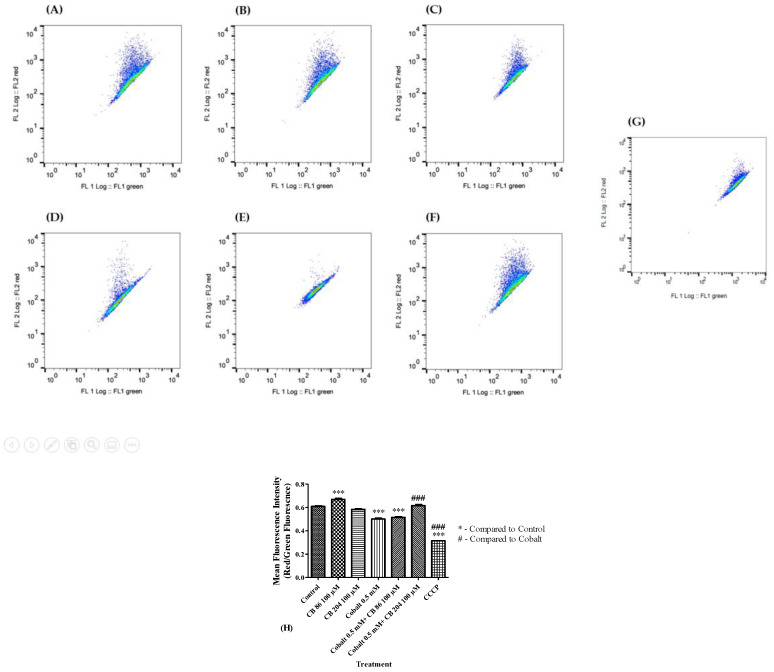
Comparison of the protective capacities of CB86 and CB204 in prevention of mitochondrial membrane potential depolarization. FACS was used to evaluate the Δψm depolarization in (**A**) Control group, (**B**) cells pretreated with CB86 alone, (**C**) cells pretreated with CB204 alone, (**D**) cells exposed to 0.5 mM of CoCl_2_, (**E**) cells pretreated with CB86 and exposed to CoCl_2_, (**F**) cells pretreated with CB204 and exposed to CoCl_2_, (**G**) positive control group exposed to CCCP, and (**H**) representative bar graph of the raw data. Results are expressed as mean ± SEM (*n* = 7 replicates in each group). ^###^ and *** *p* < 0.001.

ΔΨm depolarization was measured by JC-1 fluorescence changes as described in the methods. Significant depolarization of the ΔΨm (18%; *p* < 0.001) occurred after exposure to CoCl_2_, as compared to the control group. Pre-treatment with CB86 at a concentration of 100 µM did not show any protective effect and ΔΨm remained significantly different from the control group (*p* < 0.001). Pretreatment with CB204 prevented completely the depolarization of the ΔΨm (*p* < 0.001) induced by CoCl_2_ (Figure 5A–G). Carbonyl cyanide chlorophenylhydrazone (CCCP) was used as a positive control and its administration resulted in a significant increase in depolarization of ΔΨm (by 48% vs. control raw level; *p* < 0.001) (Figure 5H).

### 3.6. LDH Assay of Cell Death

**Figure 6:**
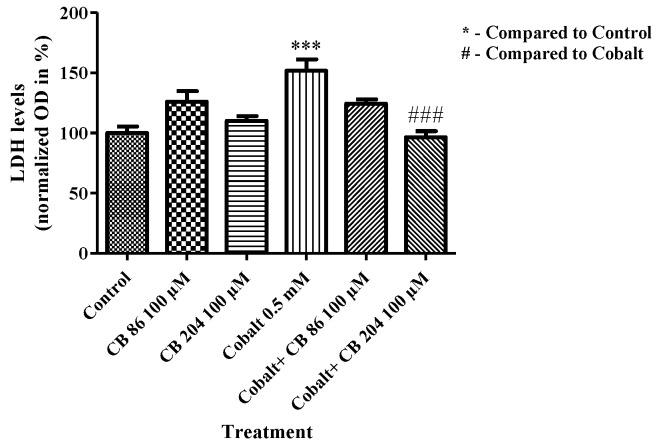
Prevention of CoCl_2_-induced cellular cytotoxicity by TSPO ligands. LDH cytotoxicity assay was performed to measure cytotoxicity levels following exposure to CoCl_2_ with or without TSPO ligands pretreatment. Results are expressed as mean ± SEM (*n* = 5 replicates in each group). ^###^ and *** *p* < 0.001.

Cytotoxicity levels were measured by release of LDH enzyme into the media. CoCl_2_ exposure resulted in a significant elevation in cytotoxicity levels by 52% (*p* < 0.001) as compared to the control group (absolute LDH level measured by OD was 0.5). Pretreatment with the TSPO ligand CB86 at a concentration of 100 µM did not attenuate significantly the LDH elevation, while CB204 at a concentration of 100 µM completely prevented the LDH elevation (100%; *p* < 0.001) as compared to the CoCl_2_-exposed group. Both CB86 and CB204 had no effect when applied alone (Figure 6).

### 3.7. XTT Assay of Cell Viability

**Figure 7:**
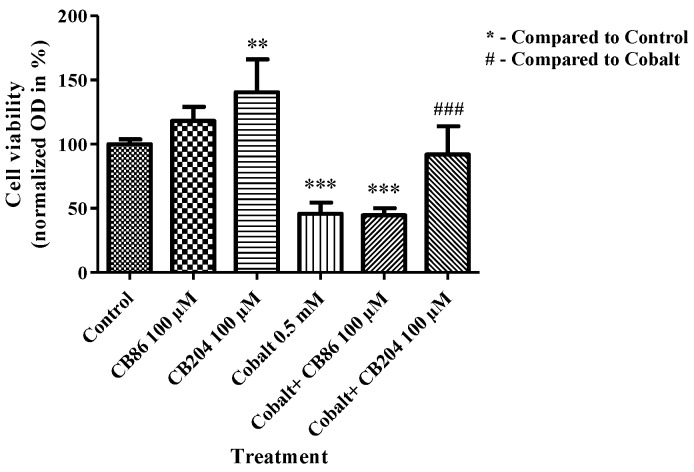
The protective effect of TSPO ligands in preventing the decrease in cellular viability. XTT assay was performed to measure the levels of cellular viability following CoCl_2_ exposure with or without TSPO ligands pretreatment. Results are expressed as mean ± SEM (*n* = 5 replicates in each group). ** *p* < 0.01, ^###^ and *** *p* < 0.001.

Following CoCl_2_ exposure, cellular viability significantly decreased by 54% (*p* < 0.001) as compared to the control group. Pretreatment with CB86 at a concentration of 100 µM did not prevent the decrease in cell viability, while CB204 at a concentration of 100 µM significantly prevented the decrease in cell viability as compared to the CoCl_2_-exposed group (85%; *p* < 0.001; Figure 7).

### 3.8. Cellular Density

**Figure 8:**
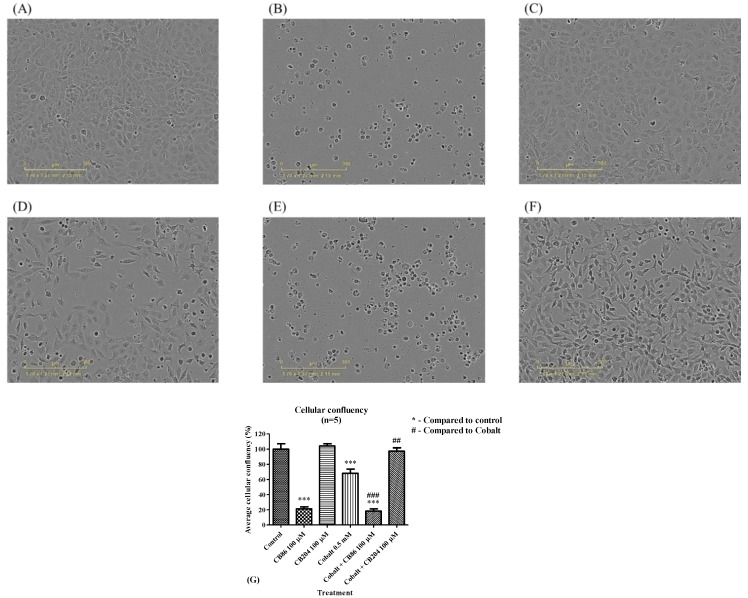
The effects of CB86 and CB204 on reductions of cell density caused by CoCl_2_. Photographs showing the density of H1299 cells in (**A**) control group, (**B**) following pretreatment with the high affinity CB86 ligand alone (100 µM), (**C**) following pretreatment with the low affinity CB204 ligand alone (100 µM), (**D**) following exposure to 0.5 mM of CoCl_2_, (**E**) following pretreatment with the high affinity CB86 at a concentration of 100 µM and exposure to 0.5 mM CoCl_2_, (**F**) following pretreatment with the low affinity CB204 at a concentration of 100 µM and exposure to 0.5 mM CoCl_2_, and (**G**) a bar graph summarizing the results. Results in Figure 8G are presented as mean ± SEM (*n* = 5 replicates in each group). ^##^ *p* < 0.01, ^###^ and *** *p* < 0.001.

Representative illustration of the alterations in cell density are depicted in Figure 8A–F. The density of the cells significantly decreased by 32% (Figure 8G) following exposure to 0.5 mM CoCl_2_ for 24 h, relative to the control group (set as 100%) (Figure 8G). The application of the high affinity ligand CB86 alone at a concentration of 100 µM induced a robust reduction in cell density (by 79%; *p* < 0.001; Figure 8G). No further decrease in cell density occurred when CB86 (100 µM) was applied as a pretreatment to CoCl_2_ exposure (by 82%; *p* < 0.001; Figure 8G). In the case of pretreatment with the low affinity ligand CB204 at a concentration of 100 µM, the density of the cells remained within the control range (Figure 8C). Also, when exposed to CoCl_2_, the pretreatment with CB204 prevented 97% of the CoCl_2_-induced decrease in cell density (Figure 8G). The collected data are summarized in Figure 8G.

In summary, CB86 (with and without CoCl_2_) caused robust reductions in cell density, while CB204 alone has no harmful effect on cell density and prevented completely reductions in cell density induced by exposure CoCl_2_.

### 3.9. Summary of the Collected Data

As the results show (Figure 3, Figure 4, Figure 5, Figure 6, Figure 7 and Figure 8), application of the high affinity TSPO ligand CB86 applied alone at a concentration of 100 µM caused: (1) Increase in the ADP/ATP ratio (Figure 3); (2) increase in ROS generation, but without effect on superoxide levels (Figure 4); (3) increase in the occurrence of ΔΨm depolarization (Figure 5); (4) increase of cell membrane disruption as assessed by LDH levels in the supernatant (Figure 6). In contrast, the low affinity TSPO ligand CB204 at the same concentration (100 µM) did not induce any cytotoxic effects (Figure 3, Figure 4, Figure 5, Figure 6, Figure 7 and Figure 8). The overall efficient protective effects indicate a favorable profile of the low affinity TSPO ligand CB204 (Figure 1, Figure 2, Figure 3, Figure 4, Figure 5, Figure 6, Figure 7 and Figure 8) with regard to efficacy and safety in hypoxic cellular conditions.

## 4. Discussion

**Scheme 2:**
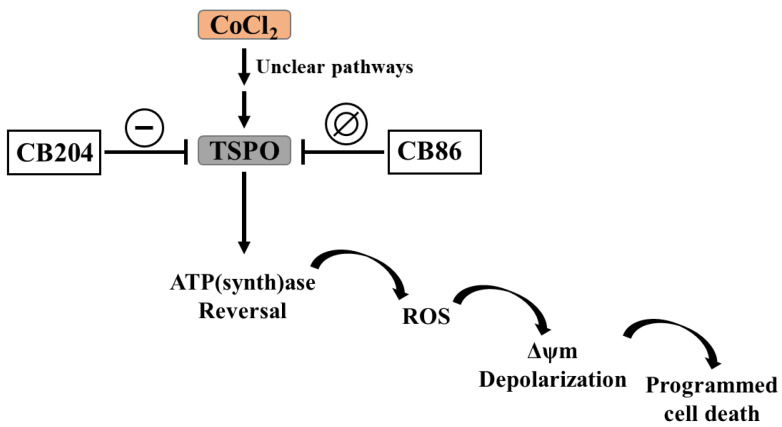
The capacity of TSPO ligand to inhibit the initiation of the CoCl_2_ cytotoxic effect and its propagation towards mitochondrial apoptosis cascade. The progression was inhibited by the low affinity CB204, but not by the high affinity CB86, TSPO ligands.

In this study, we focused on the capacity of two TSPO ligands, the high affinity CB86 and the low affinity CB204 (affinity and structure presented in Table 1 and Scheme 1) to prevent cellular damage induced by CoCl_2_ (Figure 3, Figure 4, Figure 5, Figure 6, Figure 7 and Figure 8 and Scheme 2). Based on ligand efficacy in prevention of CoCl_2_-induced cytotoxicity (Figure 1) and dose–response analysis (Figure 2), the high affinity CB86 ligand and the low affinity CB204 ligand at a concentration of 100 µM were chosen for this study. A previous study demonstrated the protective effects of the high affinity TSPO ligand PK 11195 (25 µM), in CoCl_2_-induced cell death, which was reminiscent to the protective effect of TSPO gene knockdown [22]. The protective effect of the TSPO ligands was established in several CoCl_2_-induced cytotoxic assays, including ADP/ATP ratio, ROS generation, superoxide levels, LDH cytotoxicity assay, cellular viability assay (XTT), and viable cell density. The low affinity ligand CB204 significantly inhibited the cytotoxic effect of CoCl_2_ on H1299 lung cells in all the tested assays (Figure 3, Figure 4, Figure 5, Figure 6, Figure 7 and Figure 8), furthermore, it was harmless when given alone. In contrast, the high affinity ligand CB86 was ineffective in the prevention of the cytotoxicity in most of the assays, except ROS generation. Moreover, CB86 was harmful when given alone in the assay of density of viable cells (Figure 8). Our results show that the effects of TSPO ligands reported in brain cells, are not restricted to cells of the central nervous system [35,37,38], but also applicable to lung cancer cells. It would be interesting to test whether this could be true for other types of cells as well.

The difference in the protective capacity of the two ligands could be related to differences in their affinity to TSPO or could be related to their putative agonistic or antagonistic activities at the TSPO. Several studies attempted to identify the agonistic or antagonistic properties of TSPO ligands and to sort them as agonists and antagonists. Unfortunately, such classification of TSPO ligands was abandoned, since the same ligand may exert an agonistic activity or an antagonistic activity, depending on the investigated TSPO function [48,49]. The present study confirms the observation that high affinity TSPO ligands can exert harmful, and even lethal, cellular effects, as was described in other paradigms [34,35,36]. On the other hand, most importantly, the present study supports the notion that low affinity TSPO ligands, may possess protective cellular effects and are devoid of cytotoxic effects, as was demonstrated in previous studies [35,37,38].

Different ligands have varying cellular impact in nanomolar or micromolar range concentrations depending on the TSPO complex composition and the varying affinities to the TSPO complex present in a specific cell [49]. For example, the TSPO2 isoform, the main isoform at the red blood cells membrane requires micromolar concentrations for modulation of cellular functions, while the TSPO1 isoform requires nanomolar concentrations for modulation of cellular functions [49,50,51,52]. Such difference in specific ligand-interacting structural domains, within the TSPO protein or the protein complex, could explain the variability in the impact of TSPO ligands at different concentrations in different cell types. The presence of a high affinity and a low affinity binding sites can explain the dose-dependent dual effects of some TSPO ligands [39,40,53,54]. For example, the high affinity TSPO ligands PK 11195, Ro5–4864, and FGIN-1-27 at nanomolar concentrations protect U118MG human glioma cells from ammonia-induced cytotoxicity, while at micromolar concentrations the same ligands induce cytotoxic effects [36]. We suggest that this association between affinity of the ligands to TSPO and their protective potency regarding cell viability may be the rule rather than a coincidence. Yet, this notion should be investigated further in other cell types. The two ligands CB194 and CB199 with the lowest affinity (Table 1) did not show lethal cellular effect, but they may influence other TSPO functions. It is unclear whether these ligands act at the same ligand binding site, or at different ones. At present, only a restricted number of ligand binding sites were identified [36]. Previous studies showed that binding of the ligands might be influenced by the interaction between TSPO and the TSPO-associated proteins, such as mitochondrial voltage-dependent anion channels (or mitochondrial porins; VDAC) [55,56]. For example, the anticancer drugs ErPC and ErPC3, do not bind directly to TSPO, but they exert their anti-proliferative effect indirectly through TSPO [29], while TRO 19622 binds to both the TSPO and its associated protein VDAC [57].

TSPO is associated with regulation of various intracellular and mitochondrial processes. In the current study, the cytotoxic impact of CB86 on viable cellular density was accompanied by inability to protect most of the vital cellular metabolic processes. Modulation of TSPO protein expression leads to a cascade of events, including reversal of ATP synthase activity, followed by elevated ROS generation causing oxidative stress [23,29,58,59], which is associated with ΔψM depolarization, and eventually cell death (Scheme 2) [60]. Such a correlation between TSPO and cell death, mainly apoptotic cell death, was investigated previously. Exposure of U118MG glioblastoma cells to CoCl_2_ resulted in increased apoptotic cell death, while TSPO knockdown prevented this cytotoxic effect of CoCl_2_. Moreover, exposure of the cells to the proapoptotic agent CoCl_2_ resulted in parallel increases in TSPO expression levels, as determined by Western blot and binding capacity. The same study demonstrated the role of TSPO in cellular processes leading to apoptosis [22]. Regarding another function, apart from ΔΨm collapse initiating the mitochondrial apoptosis cascade, TSPO via the mitochondria-to-cell-nucleus-pathway may also affect apoptosis induction due to modulation of cell nuclear gene expression for proteins that play a role in functional pathways regulating apoptosis [61]. Thus, the present observations on lung cells are similar to previous observations in brain cells, i.e., indicative of a common property, rather than a cell, tissue, or organ specific effect. However, research on different cell types, from different tissues and organs are required to further substantiate notion.

It was shown previously that 0.1–1 mM of CoCl_2_ can mimic hypoxia in urinary bladder smooth muscle cells as well as cardiomyocytes [62,63]. In the current study in H1299 lung cancer cell line, it was shown that the CoCl_2_-induced cascade of apoptotic events demonstrated in Scheme 2, may be prevented by TSPO ligands. The results emphasize the superiority of the low affinity TSPO ligand (CB204) in preventing cell death as well the preceding cascade of events leading to cell death as compared to the high affinity ligand (CB86). Previous studies have shown that TSPO ligands affect also cell proliferation, hence, it would also be interesting to test this for CB86 and CB204. It may be assumed that different binding domains modulate different TSPO functions.

In conclusion, our study demonstrated the robust efficacy of the low affinity TSPO ligand (CB204) in the prevention of CoCl_2_-induced cellular damages in lung cells. The protective effect of the low affinity ligand was reflected in the inhibition of the appearance of classical makers of cytotoxicity assessed in this study. In contrast, the high affinity ligand (CB86) was effective only in the prevention of oxidative stress (ROS generation). These results may have clinical implications, namely, the potential use of the low affinity TSPO ligand CB204, but not the high affinity ligand CB86, for the treatment of lung cell pathologies related to hypoxia, such as pulmonary ischemia and COPD.

Future studies should address the following issues: the impact of TSPO knockdown and other TSPO ligands, to clarify further the role of TSPO in the CoCl_2_-related cellular damage as well as the role of the TSPO ligands, preferably low affinity ligands, in the protection from non-CoCl_2_-related hypoxic damage. Noteworthy, the current study, as well as a similar previous study on U118MG glioblastoma cells, were conducted on cells from human origin. Thus, it would be of interest to investigate the effects of TSPO ligands on cells of other species. The mechanism underpinning the impact of CoCl_2_ on TSPO remains unclear (Scheme 2), further gene expression studies must be performed to identify the cellular pathways that are involved in the inhibitory effect of TSPO on in vitro and in vivo hypoxic states.

## Figures and Tables

**Table 1:** The affinity data of translocator protein (TSPO) ligands used in the current study.

Ligand Name	CB86	CB194	CB199	CB204	Alpidem
**Molecular weight**	384	484	470	498	404
**TSPO *Ki* (nM)**	1.6	285.3	193.1	117.7	0.6
